# Inoculation with *Stenotrophomonas maltophilia* LIMN and *Enterobacter roggenkampii* LCMG Enhances Maize (*Zea mays* L.) Yield and Reduces Nitrogen Fertilizer Dependence in Nutrient-Limited Soils of Semi-Arid Regions

**DOI:** 10.3390/microorganisms14092006

**Published:** 2026-09-10

**Authors:** Odilón Gayosso Barragán, Griselda Chávez Aguilar, Deli Nazmín Tirado González, Roberto Reynoso Santos, Ismael Fernando Chávez Díaz, Lily Xochilt Zelaya-Molina, Gustavo Tirado Estrada, Luis Yobani Gayosso Rosales

**Affiliations:** 1 Centro Nacional de Investigación Disciplinaria en Agricultura Familiar, Instituto Nacional de Investigaciones Forestales, Agrícolas y Pecuarias, Carretera Ojuelos-Lagos de Moreno km. 8.5, Ojuelos de Jalisco C.P. 47540, Jalisco, Mexico; gayosso.odilon@inifap.gob.mx; 2 Departamento de Ingenierías, Instituto Tecnológico El Llano Aguascalientes, Tecnológico Nacional de México, Carretera Aguascalientes-San Luis Potosí km. 18.5, El Llano C.P. 20330, Aguascalientes, Mexico; gustavo.te@llano.tecnm.mx; 3 Campo Experimental Centro de Chiapas, Instituto Nacional de Investigaciones Forestales, Agrícolas y Pecuarias, Carretera Ocozocoautla-Cintalapa km. 3, Ocozocoautla C.P. 29140, Chiapas, Mexico; reynoso.roberto@inifap.gob.mx; 4 Centro Nacional de Recursos Genéticos, Instituto Nacional de Investigaciones Forestales, Agrícolas y Pecuarias, Boulevard de la Biodiversidad 400, Rancho las Cruces, Tepatitlán de Morelos C.P. 47600, Jalisco, Mexico; chavez.fernando@inifap.gob.mx (I.F.C.D.); zelaya.lily@inifap.gob.mx (L.X.Z.-M.); 5 Campo Experimental Valles Centrales de Oaxaca, Instituto Nacional de Investigaciones Forestales, Agrícolas y Pecuarias, Melchor Ocampo 7, Santo Domingo Barrio Bajo, Villa de Etla C.P. 68200, Oaxaca, Mexico; gayosso.luis@inifap.gob.mx

**Keywords:** plant growth-promoting rhizobacteria, nitrogen use efficiency, sustainable agriculture, semi-arid soils, biological nitrogen fixation

## Abstract

Excessive nitrogen (N) fertilization in maize (*Zea mays* L.) production causes environmental degradation, particularly in semi-arid regions where high chemical inputs are necessary to maintain crop production. Plant growth-promoting rhizobacteria (PGPR) could reduce the optimal N fertilization doses. This investigation aimed to isolate bacteria, screen them for plant growth-promoting traits, and evaluate their potential effects on maize grain under different chemical N fertilization doses. Isolates *Stenotrophomonas maltophilia* LIMN and *Enterobacter roggenkampii* LCMG demonstrated multifunctional growth-promoting traits *in vitro*, including N_2_ fixation, indole-3-acetic acid and siderophore production, potassium solubilization, and desiccation tolerance, with *E. roggenkampii* LCMG also solubilizing inorganic phosphate. A field trial across four chemical N fertilization rates (0, 40, 80, and 120 kg N ha^−1^) revealed that single bacterial inoculation without N fertilizer matched or exceeded uninoculated controls receiving up to 80 kg of N ha^−1^ (the standard recommendation). Specifically, *S. maltophilia* LIMN combined with the recommended rate of 80 kg N ha^−1^ achieved the maximum grain yield (4601.2 kg DM ha^−1^), outperforming uninoculated controls (without PGPR) receiving excessive fertilization at 120 kg N ha^−1^ (3760.4 DM ha^−1^). Although maize plants benefited from inoculation with *S. maltophilia* LIMN or *E. roggenkampii* LCMG, achieving similar or better yields than crops fertilized with high chemical N inputs under stress factors such as deficiency of moisture, low-nutrient soils, and high temperatures, the present study did not test the pathogenicity and biosecurity of *S. maltophilia* or *E. roggenkampii* in crops; therefore, the results are not a direct recommendation of their use as biofertilizers before novel studies to assess all the limitations and the potential to reduce N dependency in order to design novel sustainable strategies for crop production.

## 1. Introduction

The increasing global population, projected to reach 10 billion by 2050, demands more food production [1], especially crops like maize (*Zea mays* L.), which is vital for human and livestock consumption in developing countries. However, intensive crop production has caused environmental and social problems, including chemical pollution, deforestation, biodiversity loss, and limited access to agricultural technology [2,3]. In semi-arid agricultural regions, maize grain yields are severely constrained by low organic matter content (1.0%), inherent nitrogen (N) and phosphorus (P) deficits, and erratic rainfed moisture regimes. Farmers conventionally rely on high rates of chemical nitrogen (N) fertilizers to overcome these yield barriers. However, this heavy chemical dependence causes severe agroecological degradation: excessive chemical N inputs accelerate soil acidification (pH decline) and base cation depletion, stimulate greenhouse gas emissions (specifically nitrous oxide, N_2_O), and cause widespread water pollution via nitrate (NO_3_^−^) leaching into groundwater and surface aquatic ecosystems [2,3]. At the same time, high chemical fertilizer costs yield diminishing economic returns for resource-limited dryland farmers.

Plant growth-promoting rhizobacteria (PGPR) represent a core biological mechanism for ecological intensification, offering practical opportunities to enhance agronomic nitrogen use efficiency and reduce dependence on chemical N inputs [4,5,6,7]. In the rhizosphere, PGPR enhance plant growth and stress resilience through mechanisms such as biological N_2_ fixation, phytohormone synthesis (e.g., indole-3-acetic acid, IAA), siderophore-mediated iron sequestration, and inorganic mineral solubilization, thereby promoting plant nutrient uptake [4,5,8,9,10,11,12].

Rhizobacteria can improve soil fertility [10,11,12]. Some PGPR promote plant defense against pathogens, solubilize P, and fix N_2_ [4,5,6,8,13,14]. Bacterial genera such as *Azospirillum*, *Bacillus*, *Azotobacter*, *Rhizobium*, *Enhydrobacter*, and *Pseudomonas* spp. have been widely studied to improve maize crop yields [15,16,17,18,19,20,21,22,23]. Among plant-associated rhizobacteria, *Stenotrophomonas maltophilia* and *Enterobacter roggenkampii* have emerged as promising candidates due to their multifunctional metabolic capabilities: *Enterobacter* spp. exhibit robust diazotrophic activity, suppress pathogens through specific root exudates [24], improve phosphate solubilization, and promote abiotic stress tolerance mechanisms [25,26,27]; *Stenotrophomonas* spp. strains stimulate root architectural development via substantial IAA synthesis and produce extracellular hydrolytic enzymes that facilitate nutrient cycling [28,29]. While these isolates display diverse growth-promoting traits, nitrogen remains the primary yield-limiting macronutrient and the most environmentally dynamic chemical input in dryland agriculture. Evaluating these multifunctional traits across a chemical N gradient provides a mechanistic understanding of how biological traits, such as root elongation via auxins, osmotolerance, and mineral solubilization, synergistically improve plant nutrient acquisition and support reduced chemical N dependence [30]. However, a major research gap persists regarding the field-scale validation of isolates of *S. maltophilia* and *E. roggenkampii* strains under graded chemical N inputs to determine their precise capacity to replace or reduce chemical N fertilizer requirements in maize plants grown under nutrient-depleted, rainfed semi-arid conditions.

To address this gap, this study evaluated the hypothesis that seed inoculation with isolates of *S. maltophilia* LIMN and *E. roggenkampii* LCMG reduces N fertilizer application without compromising maize crop yields. Therefore, the objective was to isolate and characterize PGPR strains and evaluate their effects on maize grain yields under N fertilizer doses in a rainfed semi-arid environment. Demonstrating the N-optimizing capacity of these strains provides critical scientific evidence for designing low-input, resilient cropping systems tailored to climate-vulnerable agricultural regions.

## 2. Materials and Methods

This study was conducted in two phases: first, in vitro characterization and molecular identification of rhizobacterial isolates, and second, a field evaluation in maize plants across multiple chemical N fertilization levels.

### 2.1. Rhizobacteria Isolation and Characterization

#### 2.1.1. Soil Sampling, Bacterial Isolation, and Morphological Characterization

The bacterial strains evaluated in this study were originally isolated and characterized during a collaborative research initiative in Chiapas, Southeastern Mexico (15°40′16″ N and 92°55′40″ W; agricultural lowlands/valleys at 500–800 masl within a regional gradient of 279–2755 masl; Köppen Aw climate classification). This is a region recognized for high microbial functional diversity [31]. Rhizosphere soil samples (0–10 cm depth) were collected directly from agroecosystems associated with maize cultivation to recover native isolates of rhizobacteria from intensively managed, low-fertility tropical soils. Specifically, *Stenotrophomonas maltophilia* LIMN was isolated from the rhizosphere of a maize–*Inga vera* agroforestry system, whereas *Enterobacter roggenkampii* LCMG was retrieved from a maize–*Canavalia ensiformis* intercropping system.

Soil samples were placed in sterile polyethylene bags, transported under cold-chain conditions (4 °C) to the laboratory, and processed immediately for bacterial isolation.

Bacterial isolation was carried out using Soil Extract Medium (SEM) agar to select for rhizosphere-adapted strains. SEM was prepared following Herrero et al. [32], where 10 g of soil was suspended in 100 mL of distilled water (1:1 *w*/*v* ratio; 0.1 g soil mL^−1^ water), stirred continuously for 1 h at 22 °C under aseptic conditions, filtered through Whatman No. 1 paper, and amended with 15.0 g L^−1^ agar (pH 7.0 ± 0.2).

For bacterial extraction, 2.0 g of root-adherent rhizosphere soil (tightly attached to the active maize root system after removing loose bulk soil by gentle shaking) from each sample was suspended in 18.0 mL of sterile phosphate-buffered saline (PBS; 10 mM K_2_HPO_4_/KH_2_PO_4_, 0.85% NaCl, pH 7.2). Suspensions were homogenized by vortexing for 3 min and centrifuged at 5000 rpm (2200× *g*) for 5 min to settle coarse particles. The resulting supernatant was subjected to 10-fold serial dilutions (10^−1^ to 10^−6^) in sterile PBS. Aliquots of 100 µL from dilutions 10^−4^, 10^−5^, and 10^−6^ were spread-plated in triplicate onto SEM agar plates under aseptic conditions. Plates were incubated at 30 ± 1 °C for 24–48 h. Distinct single colonies were purified and morphologically characterized based on standardized colony size, margin, elevation, pigmentation, texture, consistency, and optical light reflection on Luria–Bertani (LB) agar (10 g L^−1^ tryptone, 5 g L^−1^ yeast extract, 10 g L^−1^ NaCl, 15 g L^−1^ agar; pH 7.2) after 24–48 h at 30 °C. Pure cultures were preserved in 20% *v*/*v* glycerol broth at −80 °C.

To validate the broader applicability of these bioinoculants, we aimed to test their cosmopolitan potential to promote plant growth outside their native geographic origin, evaluating these strains in the semi-arid, low-fertility highland natural environment of Ojuelos, Jalisco, that served as a rigorous stress to test the potential agro-biotechnological transfer in a contrasting agroecosystem where rainfed maize faces severe compound stress driven by a critical water deficit (48 to 64% below optimal requirements) synergistically combined with high temperatures (>32 °C) and poor soil quality (<1% organic matter), which drastically restricts crop development and yield potential (SMN, CONAGUA, see Data Availability Statement).

#### 2.1.2. Plant Growth-Promoting Traits *In Vitro* and Strain Selection Criteria

Bacterial strains were isolated and characterized at the National Center for Genetic Resources (CNRG) of the National Institute for Forestry, Agriculture and Livestock Research (INIFAP). Pure cultures were maintained on the Luria–Bertani (LB) medium (10 g L^−1^ yeast extract, 5 g L^−1^ peptone, 10 g L^−1^ NaCl, 15 g L^−1^ agar). Candidates were subjected to comprehensive *in vitro* screening across six specific plant growth promotion traits.

Growth capability was assessed on the N-free Winogradsky medium (10.0 g L^−1^ glucose, 0.5 g L^−1^ K_2_HPO_4_, 0.2 L^−1^ MgSO_4_ ∙ 7 H_2_O, 0.2 g L^−1^ NaCl, 0.1 g L^−1^ MnSO_4_ ∙ 4H_2_O, 0.01 g L^−1^ FeSO_4_ ∙ 7 H_2_O, 15.0 g L^−1^ agar; pH 7.2). Isolate cultures were streak-plated and incubated at 30 ± 1 °C for 7 days; sustained growth across three consecutive subcultures on N-free agar was scored based on positive potential diazotrophic or oligonitrophilic capacity (+).

Inorganic phosphate (PO_4_^3−^) solubilization was assessed on the NBRIP agar medium containing insoluble tricalcium phosphate [Ca_3_(PO_4_)_2_, 5.0 g L^−1^]. Plates were spot-inoculated with 10 µL of log-phase bacterial culture (approx. 10^8^ CFU mL^−1^) and incubated at 30 ± 1 °C for 5 days. Phosphate solubilization was detected by visible clear halo zones surrounding colonies.

Potassium (K) solubilization was screened on the modified Alexandrov agar medium (10.0 L^−1^ glucose, 0.5 g L^−1^ MgSO_4_ ∙ 7H_2_O, 0.1 g L^−1^ CaCO_3_, 0.005 g L^−1^ FeCl_3_, 2.0 g L^−1^ Ca_3_(PO_4_)_2_, 15.0 g L^−1^ agar; pH 7.2) supplemented with potassium aluminosilicate (5.0 g L^−1^, feldspar powder) as the insoluble K source. Soluble salts were evaluated in parallel as soluble controls (KCl and KNO_3_). Spot-inoculated plates were incubated at 30 ± 1 °C for 5 days, and clear halo zones resulting from potassium mineral dissolution were measured.

Indole production (IAA) was quantified colorimetrically using the Salkowski reagent. Isolates were cultured in Trypticase Soy Broth (TSB) supplemented with 0.1% *w*/*v* L-tryptophan at 30 ± 1 °C for 48 h under continuous shaking (150 rpm). The cell-free supernatant obtained via centrifugation (10,000× *g*, 10 min) was mixed with the Salkowski reagent (0.5 M FeCl_3_ in 35% H_2_SO_4_) at a 1:2 *v*/*v* ratio and incubated in the dark at 25 °C for 30 min. Color development was quantified spectrophotometrically at 530 nm against a standard curve of pure IAA (µg mL^−1^). For semi-quantitative screening on agar plates, Trypticase Soy Agar (TSA) supplemented with L-tryptophan was flooded with Salkowski reagent, and the resulting pink halo zones were measured.

Siderophore production was evaluated using Chrome Azurol S (CAS) analytical agar plates. Spot-inoculated CAS agar plates were incubated at 30 ± 1 °C for 5 days in the dark. Siderophore production was confirmed by a distinctive color transition from blue to orange-yellow surrounding the bacterial colonies.

To quantify halo-forming efficiency [33], solubilization and production capabilities were mathematically expressed using the Relative Index (RI), evaluated as the Relative Solubilization Index (RSI) or Relative Production Index (RPI) (Equation (1)):

(1) $$RSI\;or\;RPI=\frac{colony\;diameter+diameter\;of\;halo\;zone}{colony\;diameter}$$

Desiccation and osmotic stress tolerance were evaluated on TSA plates supplemented with sucrose at 3%, 8%, 10%, and 20% *w*/*v* to induce graded hydric potentials. Radial colony extension (cm) was recorded after 48 h of incubation at 30 ± 1 °C.

Isolate selection for molecular identification and field trials was based on a cumulative multi-trait scoring system across all six screening criteria. Strains demonstrating high multifunctional PGP profiles (simultaneous diazotrophy, auxin production with RPI ≥ 1.2, siderophore production with RPI ≥ 1.7, mineral solubilization, and radial growth under ≥10% sucrose osmotic stress) were prioritized as candidates displaying the highest plant growth-promoting potential.

#### 2.1.3. Molecular Identification

The molecular identification of the bacterial strains with the highest potential for plant growth promotion was performed through phylogenetic analysis of the 16S rRNA gene. For this purpose, genomic DNA was extracted from the strains, and the 16S rRNA gene was amplified according to Barragán-Nava et al. [34]. Genomic DNA extraction and 16S rRNA gene sequencing were performed by Macrogen Inc. (Seoul, Republic of Korea) using its Standard Sequencing Service. The obtained sequences were visually inspected and edited using BioEdit version 7.2.5. A set of taxonomically related type-strain sequences was retrieved by performing a BLAST (version 2.17.0, NCBI, Bethesda, MD, USA) search. All sequences were aligned using the ClustalW algorithm implemented in MEGA X, which was also used to perform the phylogenetic analysis. The phylogenetic tree was constructed using the Maximum Likelihood method with 1000 bootstrap replicates to assess the statistical support of the tree nodes. Phylogenetic analysis of the 16S rRNA gene confirmed the taxonomic affiliation of the selected strains. Strain LCMG clustered within the genus *Enterobacter* and showed the closest relationship to *Enterobacter roggenkampii* (GenBank: OM060606.1), whereas strain LIMN clustered within the genus *Stenotrophomonas* and was most closely related to *Stenotrophomonas maltophilia* (GenBank: OM060618.1). The Maximum Likelihood tree, reconstructed under the Tamura–Nei + G + I substitution model, showed high bootstrap support for the major clades, supporting the genus-level identification of both isolates.

### 2.2. Field Evaluation in Maize

#### 2.2.1. Study Site and Environmental Conditions

The fields were situated in Ojuelos de Jalisco, Jal. (National Center for Disciplinary Research in Family Agriculture (CENID AF), INIFAP), north-central Mexico, located between 21°33′ N and 101°02′ W, corresponding to a semi-arid zone classified as BSh (hot semi-arid) (Köppen classification [31]), with a sandy soil type of low fertility. From June to October 2023, the median, maximum, and minimum temperatures were 25.84, 32.03, and 13.22 °C, with total rainfall of 286 mm irregularly distributed over the five months, according to governmental databases obtained from two nearby meteorological stations (SMN, CONAGUA, see Data Availability Statement).

#### 2.2.2. Soil Physical and Chemical Analysis

The topography is typically characterized by valleys and gentle hills, with soils classified as Haplic Xerosols (associated with Lithosols and Eutric Planosols) and Haplic Phaeozems, with less than 1% of organic matter [35]. However, soil samples from the designated maize fields were tested.

According to NOM-021-RECNAT-2000 [36], soil texture was analyzed using the Bouyoucos hydrometer method, while soil organic matter (SOM) was determined by the oxidation of organic carbon [37]. The N content and available P were analyzed using the micro-Kjendahl [38] and Bray 1 methods [39]. The contents of calcium (Ca^2+^), magnesium (Mg^2+^), potassium (K^+^), and sodium (Na^+^) were extracted by saturating the cation-exchange sites using a 1 N ammonium acetate solution (NH_4_C_2_H_3_O_2_, pH 7.0) [40]. Iron (Fe) and manganese (Mn) contents were evaluated using the diethylenetriaminepentacetic acid (DTPA) extraction method [41]. Additionally, boron (B) content was analyzed using the azomethine-H method [42], and sulfur (S) content was determined according to NOM-021-RECNAT-2000 [36]. The cation-exchange capacity (CEC) was calculated from the sum of extracted exchangeable cations (Ca^2+^, Mg^2+^, K^+^, and Na^+^) [43].

The sandy loam soils (Table 1) had a pH of ~7.5, indicating moderately alkaline conditions. These soils were characterized by low contents of SOM, N, available P, Na, and Cu; medium contents of Ca, Mg, and S; and high contents of K, Mn, and B. The FC had typical values for sandy loam soils with low water retention that limits water availability for plant roots and a PWP. Arenosol/Psamment soil has a sandy texture and rapid drainage.

After integrating the results of the soil analysis, the site was selected for its contrasting natural environment and the presence of the necessary stress conditions to test PGPR: extremely low organic matter (0.89%), poor water retention (field capacity of 21.10%, permanent wilting point of 10.80%), high bulk density (1.57 g cm^−3^), slightly alkaline pH (7.58), limited chemical fertility (very low available nitrogen (4.38 ppm), low phosphorus (3.17 ppm), apparent potassium at 463 ppm, iron at 38.41 ppm, and boron at 2.40 ppm) and reduced cation exchange capacity (7.44 meq/100 g with a base saturation of 28.69%).

#### 2.2.3. Plant Material

The tri-lineal intermediate/late hybrid H-391 (INIFAP, Mexico) adapted to the Central–East México regions was used in this study. This hybrid exhibits resistance to root and stalk lodging, good cob health, and a strong overall plant appearance, with resistance to common rust (*Puccinia sorghi*) and downy mildew (*Sclerophthora macrospora*) [44].

#### 2.2.4. Experimental Design and Crop Management

The field experiment was conducted in July 2023. All crops were exclusively tested under stress conditions, carefully avoiding confounding variables to accurately assess statistical differences between treatments and the controls. The experimental design was a randomized complete block design (RCBD). Each treatment was randomly assigned to plots (16 m length × 1.6 m width; 2 rows per treatment) within the four blocks (each measuring 16 × 25.6 m) located in the parcel (16 × 153.6 m; total area of 2458 m^2^). The blocks considered the variability in topography and sun orientation. The distance between rows was 80 cm, and the spacing between plants was 20 cm, resulting in a final crop density of 62,500 plants ha^−1^. Before seeding, a traditional soil management system that involved manual and minimal tillage was used to prepare the soil. After seeding, the shrubs were manually eliminated.

Table 2 shows the arrangement of the treatments. Doses of 0 (N-0), 40 (N-40), 80 (N-80), and 120 (N-120) kg of N ha^−1^ were evaluated with the inclusion of *S. maltophilia* LIMN (S), *E. roggenkampii* LCMG (E) or the mixture of S + E, or without PGPR inoculation (Control). The N source was agricultural-grade granular urea [CO(NH_2_)_2_, 46% N]. Fertilizer dosages were calculated strictly on an elemental N mass basis, corresponding to commercial urea application rates of 0 kg ha^−1^ (N-0), 86.95 kg ha^−1^ (N-40), 173.91 kg ha^−1^ (N-80), and 260.87 kg ha^−1^ (N-120). For individual experimental plots (25.6 m^2^), the equivalent commercial urea applied per plot was 0 g, 222.6 g, 445.2 g, and 667.8 g, respectively.

The selection of these N rates was informed by agronomic standards and the need to establish a functional yield response gradient. Specifically, 0 kg N ha^−1^ (N-0) serves as the unfertilized baseline control (without PGPR or chemical N) to quantify soil N contribution and the standalone biological effect of bioinoculation; the 40 kg N ha^−1^ (N-40) rate represented a sub-optimal fertilization level to evaluate bioinoculant efficacy under severe nitrogen limitation. The 80 kg N ha^−1^ (N-80) dose corresponded to the standard recommended rate for rainfed maize production. Finally, 120 kg N ha^−1^ (N-120) represented an above-recommended application rate, typical of high-input farming, allowing us to test whether bioinoculation enables yield optimization at recommended or reduced N inputs without relying on excessive chemical fertilization.

Half of the urea doses were applied to the crops immediately after sowing; the remaining doses were applied 35 days later. Chemical phosphorus (P) and potassium (K) fertilizers were intentionally omitted across all experimental units to test the intrinsic biological capability of the bacterial isolates to mobilize inherent soil P and K reserves (463.0 ppm exchangeable K; 3.2 mg kg^−1^ Bray 1 P) without chemical nutrient masking. Maize seeds were inoculated with PGPR in the field by applying a solution containing 1 × 10^8^ colony-forming units (CFU) mL^−1^ of *S. maltophilia* LIMN and *E. roggenkampii* LCMG at a rate of 1 L ha^−1^, delivered within a suitable volume of water as a carrier with a field sprayer. Control seeds were sprayed using only the water carrier without PGPR.

Given that *Stenotrophomonas maltophilia* and members of the genus *Enterobacter* encompass both plant-associated strains and opportunistic clinical lineages [26,29,45], strict biosafety measures and containment protocols were maintained throughout bacterial handling, inoculum preparation, and field application. All *in vitro* screening assays and cell suspension preparations were conducted under aseptic conditions inside certified biological safety cabinets. During the preparation of inoculant doses and field seed inoculation, safety precautions were implemented, including the use of nitrile gloves, laboratory coats, eye protection, and protective face masks to prevent direct contact, mucosal exposure, or aerosol inhalation. All biohazardous waste and contaminated materials were fully sterilized by autoclaving (121 °C for 30 min) prior to disposal.

#### 2.2.5. Yield and Agronomic Measurement

At physiological maturity of maize, when the kernels had reached their maximum dry weight and the plant had stopped translocating nutrients, five plants were sampled per treatment in each block (four repetitions), resulting in 320 samples. The following variables were measured: cob diameter (mm), using a standard Truper^®^ digital millimeter Vernier (Mexico City, Mexico); cob length (cm), using a graduated ruler measuring the distance from the base to the apex of the upper cob; grains per row, by counting the number of grains per row of random samples and calculating an average for each experimental unit; cob weight (g), recording the weight of the cob with the help of a digital scale; the number of cobs; grain yield (kg ha^−1^); the shelled cobs of each plot; and the 100-seed weight (g), by identifying at random one hundred grains of shelled cobs and recording the weight on a digital scale.

#### 2.2.6. Dry Matter Evaluation

After harvesting, the collected samples were oven-dried in a forced-air oven at 60 °C until they reached a constant dry weight (dry matter, DM).

### 2.3. Statistical Analysis

The analysis was run with SAS statistical software (Statistical Analysis System, V. 9.2, SAS Institute Inc., Cary, NC, USA) [46]. The analysis of variance (ANOVA) considered the RCBD and the fixed effects of the type of PGPR and N doses according to Equation (2). Least squares means (LsMeans) were compared with the least significant difference (LSD) test at *p* < 0.05.

(2) Y = µ + B_i_ + Bac_j_ + DN_k_ + (Bac × DN)_jk_ + ɛ_ijk_

where Y = cob diameter and length, grains/cob, cobs/plant, cob weight, 100-seed weight, and DM grain yield; B_i_ = the i-th block; Bac_j_ = the effect of the j-th inclusion of *S. maltophilia* LIMN and/or *E. roggenkampii* LCMG; DN_K_ = the effect of the k-th dose of nitrogen; (Bac × DN)_jk_ = interactions among fixed effect factors; ɛ_ijk_ = random error.

Orthogonal polynomials were used to test the linear, quadratic, and cubic effects using the regression models Y_i_ = β_0_ + β_i_T_i_ + ɛ_i_, Y_i_ = β_0_ + β_i_T_i_ + β_i_T_j2_ + ɛ_ij_, and Y_i_ = β_0_ + β_i_T_i_ + β_j_T_i2_ + β_k_T_i3_ + ɛ_ijk_, respectively (PROC REG). A Pearson’s linear correlation analysis was performed (PROC CORR).

## 3. Results

### 3.1. Bacterial Strains

**Figure 1:**
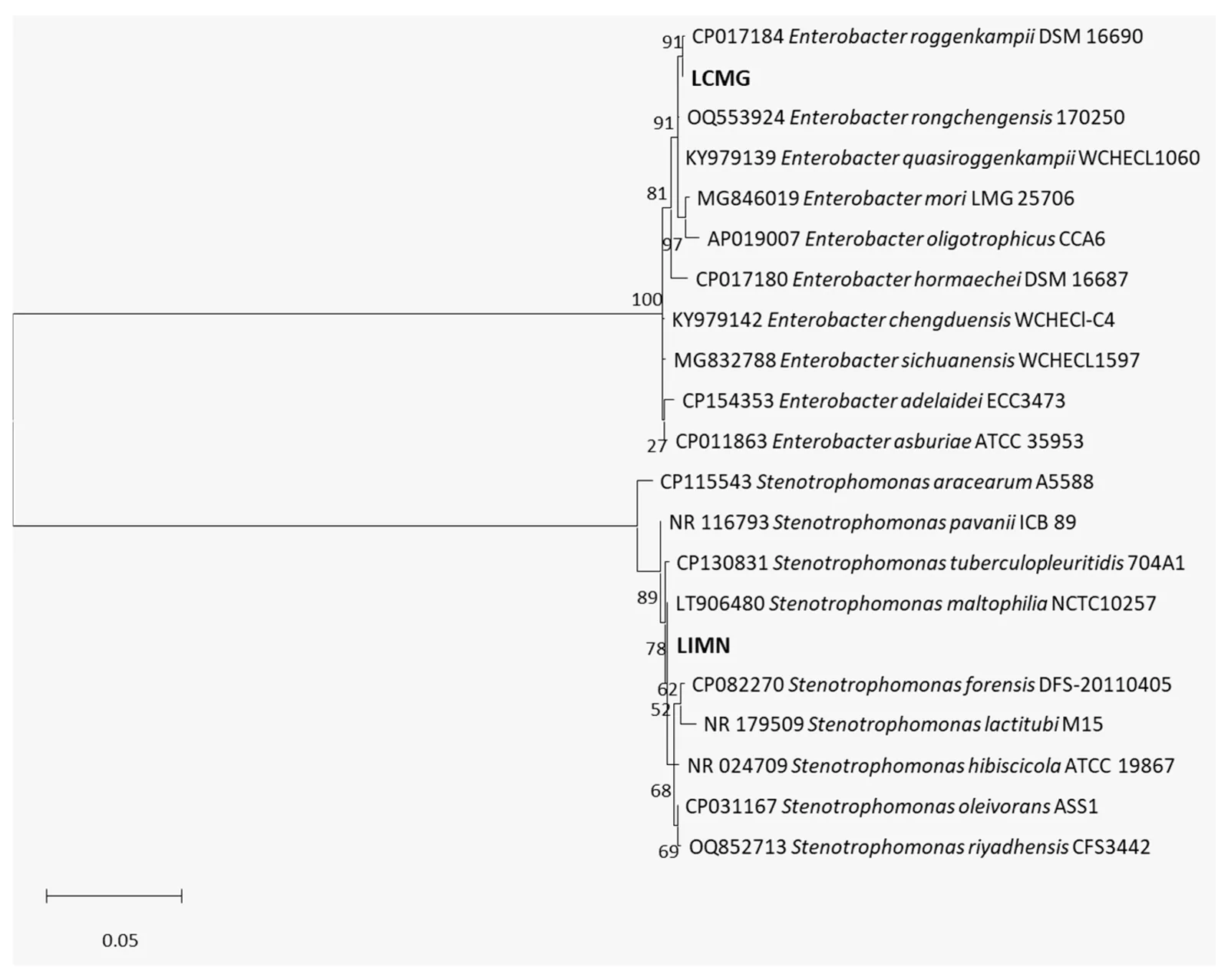
Maximum Likelihood phylogenetic tree based on 16S rRNA gene sequences; the tree was constructed in MEGA X using the Tamura–Nei model with a gamma distribution and a proportion of invariant sites (TN93 + G + I). Bootstrap support values were calculated from 1000 replicates and are shown at the nodes. The scale bar represents 0.05 nucleotide substitutions per site.

Phylogenetic analysis based on sequence comparison revealed that the isolated strains LCMG and LIMN were successfully identified at the species level (Figure 1). Strain LCMG clustered closely within the *Enterobacter* clade, showing the highest sequence identity and sharing an immediate common node with *E. roggenkampii* DSM 16690, supported by a high bootstrap confidence value of 91%. Similarly, strain LIMN was grouped within the *Stenotrophomonas* clade, forming a distinct, well-supported cluster with *S. maltophilia* NCTC 10257 (89% bootstrap support). In both cases, the negligible branch lengths separating the isolated strains from their respective reference type strains confirm their phylogenetic placement as *E. roggenkampii* and *S. maltophilia*.

### 3.2. In Vitro Plant Growth Promotion Tests

Table 3 shows that both *S. maltophilia* LIMN and *E. roggenkampii* LCMG exhibited several plant growth-promoting traits, including fixing N-free medium, producing siderophores, solubilizing K, synthesizing indoles (IAA), and growing under hydric stress. Additionally, *E. roggenkampii* LCMG was able to solubilize PO_4_^3−^ (RSI = 1.1), whereas no phosphate solubilization was detected in *S. maltophilia* LIMN.

The strain of *S. maltophilia* LIMN showed a significantly higher indole production IAA RPI than *E. roggenkampii* LCMG (1.8 *vs.* 1.2), suggesting that *S. maltophilia* LIMN might act as a PGPR with potential to stimulate cell division, subsequently promoting plant elongation. Potassium solubilization differed according to the potassium source: *E. roggenkampii* showed a significantly higher RSI with KCl (3.0 *vs.* 2.1), whereas *S. maltophilia* LIMN showed a significantly higher RSI with KNO_3_ (2.7 *vs.* 1.3). No significant difference was observed in siderophore production RPI between the two strains.

Under hydric stress conditions, *S. maltophilia* LIMN showed significantly higher (*p* < 0.05) growth than *E. roggenkampii* LCMG at 3, 8, 10, and 20% sucrose (Table 3).

### 3.3. Agronomical Variables and Grain Yield

Table 4 shows the effect of inoculating maize seeds with *S. maltophilia* LIMN and *E. roggenkampii*, combined with different N fertilization doses. Overall, cob diameter varied from 39.5 to 42.59 mm; the best averages were shown when *S. maltophilia* LIMN or *E. roggenkampii* LCMG was combined with N-0 and N-40 or when S + E was combined with N-80 to N-120 (*p* < 0.04). There were no significant differences in cob length across treatments.

The grains/row and the cob weight showed the best responses when *S. maltophilia* LIMN or *E. roggenkampii* were used without N fertilization (N-0) (averages 89.53 and 89.24 g, respectively), representing a cob weight increase of over 30% compared to the Control N-0 (68.58 g) (*p* < 0.01). However, the Control and S + E treatments had the best cob weight when the N-120 fertilization dose was applied (76.76 and 84.54 g) (*p* < 0.01).

The best grain yield was obtained through *S. maltophilia* LIMN inoculation and the N-80 fertilization dose (4601.2 kg DM ha^−1^). Although the N-80 and N-120 fertilization doses significantly improved several agronomic parameters, including the number of cobs/plants, the DM 100-seed weight, and the grain yield, which increased (*p* < 0.002); however, inoculation with *S. maltophilia* LIMN promoted better grain yield outcomes than Control, *E. roggenkampii*, and S + E treatments. Specifically, *S. maltophilia* LIMN provided a 31.9% yield increase over Control N-80 (3487.8 kg DM ha^−1^) and outperformed by 22.4%. Overall, *S. maltophilia* LIMN provided an average yield improvement of 19.2% over the Control across fertilization levels (*p* < 0.003).

### 3.4. Trends of Grain Yield Across Doses of N Fertilization and PGPR Inoculation and Correlations

Regardless of the effect of PGPR inoculation, the highest cob weights were shown in treatments without N fertilization (Table 5); however, these variables had quadratic or cubic trends, with the worst when 40 to 80 kg N ha^−1^ was added (*p* < 0.05).

The number of cobs/row, the grain yield, and the 100-seed weight had quadratic effects among the N fertilization doses (*p* < 0.01); therefore, 40 and 80 kg N ha^−1^ were the optimal fertilization doses.

The individual application of the rhizobacteria *S. maltophilia* LIMN or *E. roggenkampii* LCMG, as well as their combined inoculation (Table 6), improved yield compared to those treatments without PGPR (Control) (*p* < 0.05).

The treatments S + E and N-0 promoted grain yields similar to Control with N-120. Linear effects of N fertilization doses were observed in the Control and when seeds were inoculated with the S + E mixture (*p* < 0.05); therefore, for those treatments, the best grain yields were observed when N-120 was applied (3760.42 and 4546.97 kg DM ha^−1^). However, where maize seeds were inoculated individually with either *S. maltophilia* LIMN or *E. roggenkampii* LCMG, N fertilization doses had cubic trends (*p* < 0.01); for these treatments, the optimal fertilization dose was found to be N-80 (4601.19 and 4134.23 kg DM ha^−1^), which represents gains of 31.9% and 18.5% over the Control at the same fertilization level.

**Figure 2:**
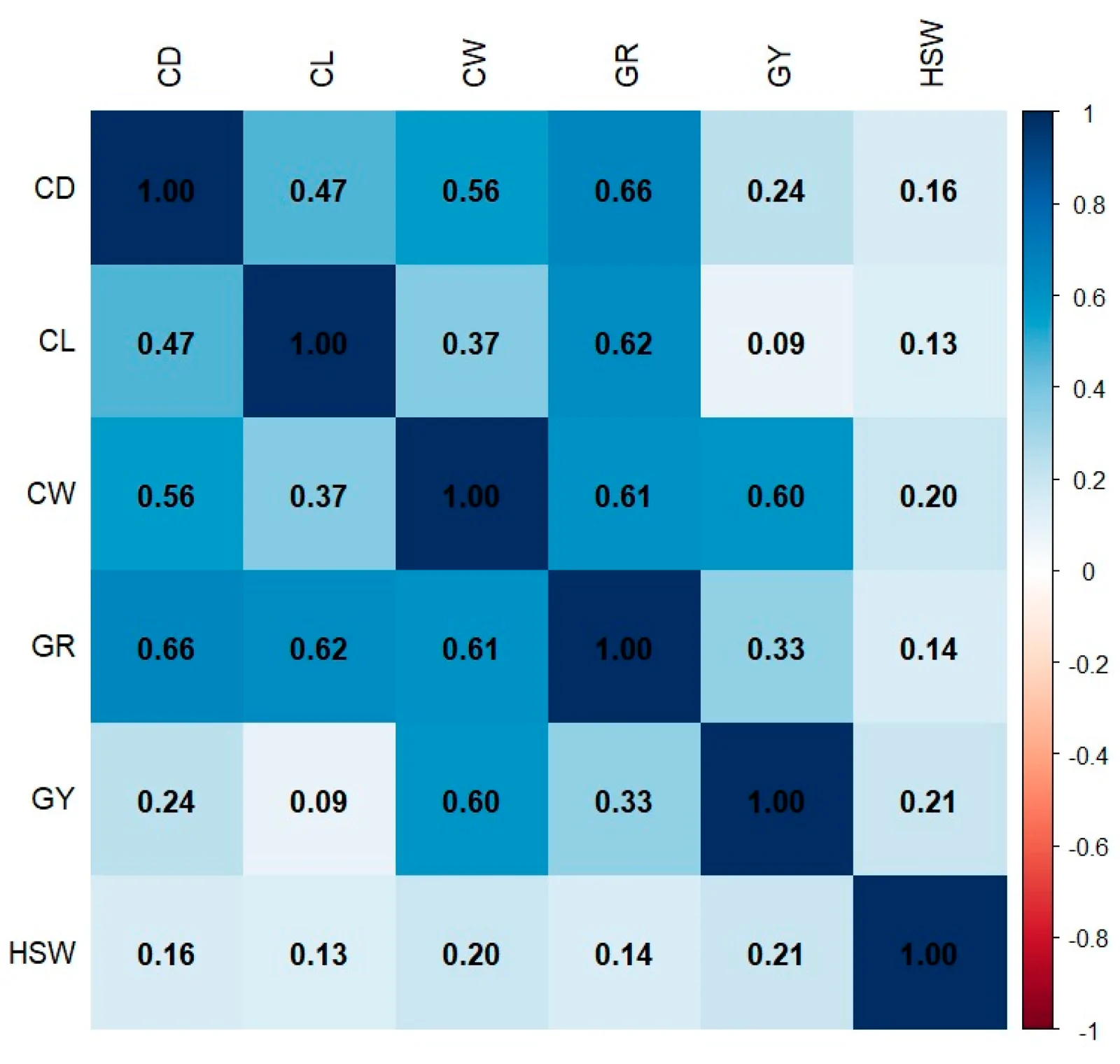
Pearson’s correlation among physio-technical and grain yield variables in maize crops fertilized with N and/or treated with plant growth-promoting rhizobacteria. CD: cob diameter, CL: cob length, GR: grains/row, CW: cob weight, GY: grain yield, and HSW: 100-seed weight.

Figure 2 shows the simple correlations between agronomic and yield variables. The number of grains/row (GR) strongly correlated with cob diameter (CD) and length (CL) (*r =* 0.66 and 0.62; *p* < 0.0001). However, the cob weight (CW) correlated with the CD and the number of GR (*r* = 0.56 and 0.61; *p* < 0.0001).

Additionally, CW and cobs per plant significantly correlated with grain yield (*r* = 0.60 and 0.57; *p* < 0.0001).

## 4. Discussion

### 4.1. Natural Crop Stress Conditions

The experimental sites were intentionally selected due to their diverse natural stress conditions for maize cultivation. Effective soil management to improve limited SOM, N, and P conditions includes chemical fertilizers, organic amendments such as composts, and inoculation with beneficial rhizobacteria [47]. The environmental conditions of the present study represented an extreme, compound abiotic stress scenario for rainfed maize, driven by the synergy of three severe limiting factors: The primary constraint is a critical water deficit, with recorded precipitation at 286.19 mm (up to 64% below optimal requirements), exacerbated by a sandy soil characterized by poor moisture retention and high bulk density (1.57 g cm^−3^). This drought condition is compounded by thermal stress from average maximum temperatures of 32.03 °C (peaking above 35 °C), which significantly accelerates plant desiccation and reduces the pollen viability. Additionally, the crop faces severe nutritional stress due to highly degraded soil fertility, marked by critically low organic matter (0.89%), severe N (4.38 ppm) and P (3.17 ppm) deficiencies, and a reduced overall nutrient retention capacity. Under normal circumstances, the study area is considered unsuitable for maize farming due to the synergistic impact of drought, extreme heat, and soil degradation diminishing crop yields by 40% to 60%, with even more drastic losses expected if these limitations coincide with the critical flowering stage.

Therefore, inoculation of plants with PGPR increased the plant’s root volume, nutrient uptake, modulation of hormones, and accumulation of stress-related metabolites compared with the controls. In the present study, as in others, PGRP represented an effective strategy to improve crop growth and yield [6,11,12,13,20,48,49,50].

### 4.2. Role of PGPR in Alleviating Field Stress Conditions

The superior agronomic performance observed for *S. maltophilia* LIMN and *E. roggenkampii* LCMG must be interpreted within the compound abiotic stress regime characterizing the experimental environment. Maize production in the study area faced a severe hydrological deficit; under such drought and heat stress, bacterial synthesis of auxins (such as IAA) plays an essential physiological role by stimulating lateral root formation and elongation, thereby expanding the active root surface area and enhancing water and nutrient acquisition from moisture-depleted soil horizons [30].

PGPR increased N availability [18,20,27,51]. Kuan et al. [52] showed that inoculating maize with PGPR strains significantly increased plant N uptake, dry biomass, and yield. In the present study, grain yields were increased when *S. maltophilia* LIMN and *E. roggenkampii* LCMG were applied, reducing the optimal dose of N fertilization, showing that PGPR can be as effective as high doses of N fertilization [53,54,55]; additionally, PGPR improved the crop adaptation to water-limited conditions [6,9,56] and high temperatures [20,57]. Therefore, inoculation with PGPR might be a sustainable alternative treatment to mitigate soil degradation and water pollution [58,59,60], and even to improve soil fertility and the composition of the grain’s proteins [61]. In environments with drought, high temperatures, and soil salinity, PGPR can enhance crop tolerance to abiotic stress [10,11,12,13,62,63,64].

Chen et al. [65] found that inoculating maize with *Sinorhizobium* sp., *Bacillus* sp., *Sphingomonas* sp., and *Enterobacter* sp. strains significantly increased the amount and diversity of beneficial bacteria in the rhizosphere; these changes were positively correlated with a 29% increase in maize grain yield. Similarly, Maulina et al. [66] evaluated the effects of *Glutamicibacter nicotianae*, *Brevibacillus invocatus*, and *Micrococcus luteus* in maize; they reported a significant increase in nutrient uptake, leaf chlorophyll content, net assimilation rate, and crop growth rate compared to the Control. In agreement with these observations, seed biopriming with endophytes like *Bacillus tequilensis* LRB17 and multi-strain PGPR consortia has been reported to enhance seedling vigor, soil nutrient availability, and grain yield under field conditions [16,18]. Additionally, bacterial inoculation contributes to abiotic stress amelioration, such as salinity tolerance in maize [17], reinforcing its relevance for global maize production systems [2].

A critical bottleneck limits their practical adoption: commercial inoculants frequently show variable or poor performance under open-field rainfed conditions due to the poor environmental adaptability of non-adapted strains [30,59]. Isolates originating from stress-prone environments possess intrinsic physiological traits that enhance rhizosphere persistence and plant functional interactions under abiotic stress [20,56]. *S. maltophilia* LIMN and *E. roggenkampii* LCMG solubilized K, contributing to the optimized agronomic output [67]. *S. maltophilia* LIMN, which showed a higher RPI for IAA, stimulated root development and promoted plant elongation, while *E. roggenkampii*, which had a better RPI for siderophores, increased iron uptake.

In maize crops, *S. maltophilia* could increase microbial diversity, showing a synergism with the maize root exudates [25,29], enhancing the biological control of pathogenic fungi [28]. Conversely, *Enterobacter* sp. might improve maize yield [68] because of its ability to promote plant growth through various mechanisms such as N fixation, P solubilization, and the biocontrol of plant pathogens [24,26].

Siderophores are critical for microorganisms to uptake iron in the form of siderophore–Fe^3+^ complexes and then translocate it into the cell cytosol, improving agricultural production in iron-deficient soils; siderophores can act as metal-chelating agents to regulate the availability of Fe^3+^ inside the plant rhizosphere [62,69,70]. Furthermore, IAA is an essential plant auxin that plays a key role in root formation and promotes root hair growth, allowing plants to access more water and nutrients from the soil, which is particularly important during drought conditions [57,63]. Together, these mechanisms explain the superior performance of *S. maltophilia* LIMN observed in this study. Multi-strain inoculation could show synergistic effects [71,72] on maize crops [16,17], wheat [73], barley [74], and canola [50,72,75]. However, specific strain mixtures must be considered to avoid negative interactions. In this study, the co-inoculation of *S. maltophilia* LIMN and *E. roggenkampii* LCMG (S+E) did not show additive or synergistic effects on grain yield compared to single inoculations [1]. This lower performance of the mixed inoculum could be attributed to competitive exclusion in the rhizosphere, where both strains compete for limited root exudates and spatial attachment sites on the root surface [30,56]. Additionally, potential signal interference or metabolic antagonism between the two strains may have suppressed their individual growth-promoting capabilities [71].

### 4.3. Evaluation of Pathogenicity and Biosafety Limitations Prior to Field Scaling

A critical consideration regarding the agricultural deployment of *Enterobacter* and *Stenotrophomonas* species relates to biosafety and opportunistic human pathogenicity. While environmental and rhizosphere-associated strains of *S. maltophilia* and *E. roggenkampii* frequently harbor multifaceted plant growth-promoting traits [26,29], clinical lineages within these taxonomic groups are recognized opportunistic pathogens capable of causing nosocomial infections in immunocompromised individuals [45,76,77]. Consequently, direct translational adoption or commercial formulation of these bacteria as biofertilizers must be approached with caution until their biosafety has been rigorously established at the strain level.

Another important concern about the bacterial strains evaluated in this study is their taxonomic identity. *Stenotrophomonas maltophilia* and members of the genus *Enterobacter* include environmental and plant-associated bacteria, but these groups also contain strains with opportunistic pathogenic potential [45,76,77]. Kumar et al. [29] highlighted the considerable diversity within *S. maltophilia*, emphasizing the need to distinguish plant-associated strains from clinically relevant lineages based on strain-specific characteristics. A similar consideration applies to *E. roggenkampii*. Mukharjee et al. [78] reported considerable genetic diversity among *E. roggenkampii* isolates, with environmental and clinical strains occurring in distinct phylogenetic groups, emphasizing the importance of evaluating characteristics at the strain level. Therefore, identification as *E. roggenkampii* alone does not establish the biosafety of a strain. In the present study, *S. maltophilia* LIMN and *E. roggenkampii* LCMG were isolated from maize rhizosphere soils and subsequently evaluated for their effects on maize under field conditions. Although both strains showed plant growth-promoting traits and affected maize grain yield, these results do not establish their biosafety or exclude strain-specific pathogenic potential. Future investigations should incorporate whole-genome sequencing (WGS) and genomic screening for virulence-associated determinants, antimicrobial resistance (AMR) genes, and mobile genetic elements [26,45,76,77]. Therefore, the observed agronomic effects should be interpreted independently from the biosafety status of these strains, and further strain-level characterization would be required before considering broader agricultural application.

Finally, two main technical limitations of this study should be noted. First, in situ bacterial viability and rhizosphere colonization efficiency were not directly assessed post-inoculation. Second, the field trial was restricted to a single experimental site during one rainfed growing season. Although the observed yield improvements and chemical N reductions demonstrate the functional effects of the inoculated strains under the conditions evaluated, multi-year and multi-location field trials under contrasting soil and climatic conditions are required to account for inter-annual weather fluctuations and determine the consistency of the observed responses. Future research should also incorporate quantitative molecular tools to monitor the persistence, abundance, and rhizosphere colonization efficiency of the inoculated strains under variable field environments.

## 5. Conclusions

The present results are not a direct recommendation of using *S. maltophilia* or *E. roggenkampii* as biofertilizers; studies are first required in order to test variables related to their biosecurity during crop management and after harvest. However, the individual use of *S. maltophilia* LIMN and *E. roggenkampii* LCMG, as well as the mixture of S + E without N fertilization, had similar or superior effects on grain yield to the Control with 80 to 120 kg N ha^−1^; furthermore, *S. maltophilia* had the strongest overall effect in improving maize yields. These plant growth-promoting rhizobacteria (PGPR) demonstrated potential to optimize chemical N fertilizer requirements for maize in semi-arid regions; however, multi-strain bacterial inoculation must be carefully designed to avoid negative competitive interactions among bacteria, and their consistent efficacy requires validation across diverse production scenarios. Furthermore, the lack of direct verification of bacterial viability and colonization, combined with this study being limited to a single location and season, restricts the generalizability of the results.

## Figures and Tables

**Table 1:** Characterization of soil (physical and chemical properties) of the experimental site.

Soil Properties	Value
Textural class	Sandy
SOM (Soil organic matter)	0.89%
N (Nitrogen)	4.38 N-NO_3_^−^ ppm
Available P (Phosphorus)	3.17 ppm
Ca (Calcium)	884.08 ppm
Mg (Magnesium)	135.02 ppm
K (Potassium)	463 ppm
Na (Sodium)	38.35 ppm
Mn (Manganese)	63.59 ppm
Total CaCO_3_ (Total calcium carbonate)	0.10 ppm
S (Sulfur)	9.16 ppm
B (Boron)	2.40 ppm
Cu (Copper)	0.24 ppm
Iron (Fe)	38.41 ppm
EC (Electrical conductivity)	0.53 ds m^−1^
BD (Soil bulk density)	1.57 g cm^−3^
Base saturation	28.69%
pH (Hydrogen potential)	7.58
FC (Soil field capacity)	21.10%
CEC (Cation exchange capacity)	7.44 meq/100 g
PWP (Permanent wilting point)	10.80%

**Table 2:** Arrangement of treatments evaluated and their nomenclature.

N-Doses	Control (Without Bacteria)	N + *S. maltophilia* LIMN (S)	N + *E. roggenkampii* LCMG (E)	N + S + E
N-0 *	Control N-0	S + N-0	E + N-0	S + E + N-0
N-40	Control N-40	S + N-40	E + N-40	S + E + N-40
N-80	Control N-80	S + N-80	E + N-80	S + E + N-80
N-120	Control N-120	S + N-120	E + N-120	S + E + N-120

* N-0, N-40, N-80 and N-120: doses of fertilization of 0, 40, 80, and 120 kg of N ha^−1^.

**Table 3:** Plant growth promotion characterization *in vitro* of *Stenotrophomonas maltophilia* and *Enterobacter roggenkampii*.

Effects	*Stenotrophomonas maltophilia* LIMN	*Enterobacter roggenkampii* LCMG
PO_4_^3−^ RSI	0 ^b^	1.1 ^a^
K RSI KCl	2.1 ^b^	3.0 ^a^
K RSI KNO_3_	2.7 ^a^	1.3 ^b^
IAA (Indole production) RPI	1.8 ^a^	1.2 ^b^
Siderophore RPI	1.7 ^a^	2.0 ^a^
Bacteria growth (cm), sucrose at:		
3%	2.7 ^a^	1.4 ^b^
8%	2.0 ^a^	1.5 ^b^
10%	2.5 ^a^	1.5 ^b^
20%	1.8 ^a^	1.2 ^b^

PO_4_^3−^, phosphate; K, potassium; IAA, indole production; RSI and RPI, relative solubilization and production indexes, respectively. Different lowercase superscript letters within the same row indicate statistically significant differences between strains according to Tukey’s test (*p* < 0.05).

**Table 4:** *Stenotrophomonas maltophilia* LIMN, *Enterobacter roggenkampii* LCMG, and N doses affect agronomic variables and maize grain yield.

Bacteria	N-Doses	Cob Diameter (mm)	Cob Length (cm)	Grains /Row	Cob Weight (g)	Cobs /Plant	Grain Yield (kg DM ha^−1^)	100-Seed Weight (g DM)
Control	N-0	39.63	9.36	20.67	68.58	1.47	3134.6	23.36
	N-40	43.20	10.30	22.85	72.94	1.47	3344.8	25.31
	N-80	39.82	9.74	22.23	70.14	1.60	3487.8	24.00
	N-120	41.03	10.87	23.49	76.76	1.57	3760.4	25.91
*S. maltophilia* LIMN	N-0	42.59	10.65	24.04	89.53	1.37	3640.5	24.14
	N-40	40.95	10.07	22.30	76.79	1.57	3731.1	23.27
	N-80	41.18	10.58	23.43	88.84	1.67	4601.2	25.73
	N-120	41.42	10.48	23.50	84.47	1.67	4395.8	27.08
*E. roggenkampii* LCMG	N-0	41.94	11.19	24.86	89.24	1.30	3652.2	26.68
	N-40	39.68	9.22	21.31	77.24	1.50	3617.8	25.84
	N-80	40.64	10.42	23.49	82.46	1.60	4134.2	24.26
	N-120	40.13	9.62	21.62	69.82	1.74	3766.3	26.30
S + E	N-0	40.50	9.67	22.01	77.14	1.57	3723.7	22.95
	N-40	40.29	9.67	21.69	73.11	1.57	3586.5	23.73
	N-80	39.50	11.27	21.43	68.81	1.63	3536.5	25.37
	N-120	41.34	10.52	23.79	84.54	1.73	4547.0	27.91
SE		0.82	0.56	1.17	4.13	0.38	243.84	1.33
LSD_0.05_		1.35	0.92	1.93	6.79	0.63	399.90	2.19
CV (%)		4.90	13	12.67	12.97	12.04	15.75	12.94
*p*-values								
Bacteria		0.23	0.77	0.53	0.0003	0.25	0.003	0.68
N dose		0.43	0.35	0.61	0.22	0.0001	0.002	0.04
Bacteria × N dose		0.04	0.13	0.29	0.01	0.57	0.19	0.50

S, *S. maltophilia* LIMN; E, *E. roggenkampii* LCMG; N-doses, nitrogen doses N-0, N-40, N-80, and N-120 kg of N ha^−1^; SE, standard error; LSD, least significant differences; CV, coefficient of variation; *p*-values, probability values.

**Table 5:** Trends of agronomic and maize grain yields across N fertilization doses.

N-Doses	Cob Weight (g)	Cobs/Plant	Grain Yield (kg DM ha^−1^)	100-Seed Weight (g DM)
N-0	81.12 a	1.43 c	3537.7 b	24.28 b
N-40	75.02 b	1.53 bc	3570.0 b	24.54 b
N-80	77.56 ab	1.63 ab	3939.9 a	24.84 b
N-120	78.90 ab	1.67 a	4117.4 a	26.80 a
LSD_0.05_	5.83	0.11	343.48	1.87
Trend’s *p*-values				
Lineal	*	***	**	*
Quadratic	*	**	***	*
Cubic	*	NS	NS	NS

Different letters are statistical differences among means; *, **, and ***, significance at *p* < 0.05, *p* < 0.01, and *p* < 0.0001, respectively; NS, *p* > 0.05.

**Table 6:** Trends of maize grain yield across the *Stenotrophomonas maltophilia* LIMN, *Enterobacter roggenkampii* LCMG, and N fertilization doses.

N-Doses	Control	*S. maltophilia* LIMN (S)	*E. roggenkampii* LCMG (E)	E + S
N-0	3134.55	3640.54	3652.20	3723.66
N-40	3344.79	3731.07	3617.82	3586.46
N-80	3487.80	4601.19	4134.23	3536.46
N-120	3760.42	4395.83	3766.27	4546.97
Trend’s *p*-values				
Lineal	*	***	**	**
Quadratic	NS	**	***	NS
Cubic	NS	**	**	NS

N-doses, N fertilization: N-0, N-40, N-80, and N-120 kg of N ha^−1^; Control, without plant growth-promoting rhizobacteria (PGPR); *, **, ***, significant effects at <0.05, <0.01 and <0.0001, respectively; NS, not significant (*p* > 0.05); *p*-values, probability values.

## Data Availability

Data collected from the execution of the experimental phases and calculated databases, or a more extensive explanation about experimental designs, can be made available by direct contact with the corresponding authors [chavez.griselda@inifap.gob.mx (G. Chávez Aguilar) and deli.tg@llano.tecnm.mx (D.N. Tirado González)]. However, the overall objective behind the request might be required before sending the information. Information about bacterial strains is available at https://ageconsearch.umn.edu/record/349256/?v=pdf (accessed on 3 September 2026) and https://www.ncbi.nlm.nih.gov/nuccore/OM060618.1 (accessed on 4 September 2026). The agroclimatic databases for the study area were provided by governmental meteorological stations and remain publicly accessible on the official CONAGUA website available at https://smn.conagua.gob.mx/es/climatologia/temperaturas-y-lluvias/resumenes-mensuales-de-temperaturas-y-lluvias (accessed on 4 September 2026).

## Abbreviations

The following abbreviations are used in this manuscript:

IAA	Indole-3-acetic acid
B	Boron
CEC	Cation exchange capacity
DM	Dry matter
Fe	Iron
K	Potassium
Mg	Magnesium
Mn	Manganese
N	Nitrogen
Na	Sodium
RPI	Relative production index
RSI	Relative solubilization index
S	Sulfur
PGPR	Plant growth-promoting rhizobacteria
PWP	Permanent wilting point
